# Hypothesis on monochromatic vision in scorpionflies questioned by new transcriptomic data

**DOI:** 10.1038/s41598-018-28098-2

**Published:** 2018-06-29

**Authors:** Alexander Böhm, Karen Meusemann, Bernhard Misof, Günther Pass

**Affiliations:** 1 Department of Integrative Zoology, University of Vienna, Althanstraβe 14, 1090 Vienna, Austria; 2 Department of Evolutionary Biology and Ecology, Institute for Biology I (Zoology), University of Freiburg, Hauptstraβe 1, 79104 Freiburg, Germany; 3 Center for Molecular Biodiversity Research, Zoological Research Museum Alexander Koenig, Adenauerallee 160, 53113 Bonn, Germany; 4 Australian National Insect Collection, CSIRO National Research Collections Australia (NRCA), Acton, ACT 2601 Australia

## Abstract

In the scorpionfly *Panorpa*, a recent study suggested monochromatic vision due to evidence of only a single opsin found in transcriptome data. To reconsider this hypothesis, the present study investigates opsin expression using transcriptome data of 21 species including representatives of all major lineages of scorpionflies (Mecoptera) and of three families of their closest relatives, the fleas (Siphonaptera). In most mecopteran species investigated, transcripts encode two opsins with predicted peak absorbances in the green, two in the blue, and one in the ultraviolet spectral region. Only in groups with reduced or absent ocelli, like *Caurinus* and *Apteropanorpa*, less than four visual opsin messenger RNAs have been identified. In addition, we found a Rh7-like opsin in transcriptome data derived from larvae of the mecopteran *Nannochorista*, and in two flea species. Peropsin expression was observed in two mecopterans. In light of these new data, we question the hypothesis on monochromatic vision in the genus *Panorpa*. In a broader phylogenetic perspective, it is suggested that the common ancestor of the monophyletic taxon Antliophora (Diptera, Mecoptera and Siphonaptera) possessed the full set of visual opsins, a Rh7-like opsin, and in addition a pteropsin as well as a peropsin. In the course of evolution individual opsins were likely lost in several lineages of this clade.

## Introduction

Colour vision has two prerequisites^1,2^: receptors with different spectral responses and a neural system that can process their output in a way that preserves colour information. Ultimately the presence of colour vision in a species can only be verified by behavioural experiments. Yet, showing that photoreceptors express different opsins already gives valuable clues regarding the possibility of colour vision.

Opsins belong to the large family of G protein coupled receptors (GPCRs) which form a photoreceptive complex when covalently bound to their chromophore retinal. An incident photon causes the cis-retinal isomer to change its conformation to all-trans which leads to an interaction of the opsin with G proteins and ultimately to a de- or hyperpolarization of the receptor cell^3^. The wavelength of peak absorption depends on relatively few spectral tuning sites in the amino acid sequence of the opsin protein^4–6^. Choice of chromophore or use of special sensitizing pigments can further influence spectral sensitivity^7^. Opsins can be differentiated according to the morphology of the receptor cells they are expressed in (ciliary or rhabdomeric), the G protein they interact with, and by criteria based on gene phylogenies^8^. Another useful distinction is between visual and non-visual opsins, the latter being expressed in various tissues not directly involved in visual functions^9^. Opsin-based photoreception is universal in animals and it has been concluded that the last common ancestor of Bilateria already possessed at least nine different opsins^10^. Five of them were likely still present in the ancestral arthropod, with one being used for visual tasks^11^. This single opsin gave rise to all visual opsins observed in extant arthropods by multiple duplication events^8,12^.

Possession of multiple opsins is neither necessary nor sufficient for colour vision:^1,13^ in case only a single opsin is expressed, colour vision could still be possible by means of differential optical filtering along the light path, e.g. by screening pigments, and multiple opsins can be expressed in a single receptor to enable a broadband response. Insect vision generally is trichromatic with green, blue and UV receptors^4,7^. Yet the number of opsins, the ommatidial types and their arrangement differ^14^. For example duplication led to a total number of over 20 opsins in some dragonflies^15–17^, but it has not yet been explored how these different opsins are put to use on the receptor and neural level. Loss of visual opsin classes has been found mostly as an adaptation to special photic environments, like nocturnal, subterranean or aquatic life^18–20^. Nonetheless, a recent study^21^ recovered only a single long wavelength sensitive opsin in two scorpionfly species of the genus *Panorpa* (Mecoptera: Panorpidae) using transcriptome data. This is astonishing considering the well-developed compound eyes and ocelli in this genus and the fact that the same study found an opsin with a long- and two opsins with a short wavelength peak absorbance in the mecopteran genus *Boreus*^21^. While compound eye, ocellar and larval eye ultrastructure has been investigated in some depth^22–27^, only two studies so far dealt with opsins in scorpionflies (Mecoptera)^21,28^ and their closest relative order^29^, the fleas (Siphonaptera)^28^.

Therefore we generated transcriptomic data from the head of a female specimen of *Panorpa communis* and sampled published^29,30^ and unpublished transcriptome data generated by the 1KITE consortium (www.1kite.org). Upon screening all transcriptome data, we found four visual opsins with different predicted spectral sensitivities, expressed in almost all investigated mecopterans. Furthermore, we report opsins of unknown, likely non-visual, function in both Mecoptera and Siphonaptera.

## Methods

### Animal collection

A female *Panorpa communis* was collected in Vienna, Austria (08/06/2016). Discrimination from the very closely related species *Panorpa vulgaris*^31–33^ was based on differences in the wing pattern^34^. The specimen was kept under natural illumination for one day, decapitated under carbon dioxide anaesthesia and the head was immediately used for RNA extraction.

### RNA extraction, cDNA library preparation and sequencing

Total RNA of the whole head (including antennae) of the aforementioned *Panorpa communis* specimen was extracted using a RNeasy Plus Universal Mini Kit (Qiagen) according to the manufacturers protocol, with further cleanup by a column based kit (RNA clean and concentrator 5, Zymo Research). cDNA library preparation and next generation paired-end sequencing were performed by VBCF (Vienna Biocenter Core Facilities; NEB bead based mRNA Enrichment and Illumina HiSeq v4 reagent kit). Slightly over 12 million paired end reads with a nominal read length of 125 bp and a GC content of 37% were sequenced on an Illumina HiSeq. 2500 device.

### Sequence data processing

Access has been granted for unpublished raw sequence reads and current assemblies generated within the 1KITE project. 1KITE data will be released only upon publication of the associated phylogenetic papers but are available upon request. Unless stated otherwise in Table 1, the 1KITE project processed whole animals. All transcriptome data provided by 1KITE were sampled, sequenced and processed like previously described^35^. Transcriptome assemblies, raw sequencing data or single sequences of all other species were downloaded from NCBI. Raw sequence reads of the *Panorpa communis* head transcriptome generated for this study is deposited at the NCBI SRA archive. The NCBI accession numbers or 1KITE library identifiers for all data used in this study are listed in Supplementary Table S1.

**Table 1:** Data mining results.

Species	Family	Total opsin hits	Opsins [isoforms listed once]	Sex	Pooled individuals	Notes
**Siphonaptera**						
*Ceratophyllus gallinae*	Ceratophyllidae	0	—	n.d.	>100	
*Oropsylla silantiewi*	Ceratophyllidae	2	LwB	?	?	
*Ctenocephalides felis*	Pulicidae	1	Rh7-like	n.d.	>50	
*Tunga penetrans*	Tungidae	0	—	n.d.	several	eggs
*Archaeopsylla erinacei*	Pulicidae	2	LwB, pteropsin	n.d.	47	adults
*Xenopsylla cheopis*	Pulicidae	7	LwB, Rh7-like, pteropsin	n.d.	?	
**Mecoptera**						
*Nannochorista dipteroides*	Nannochoristidae	3	LwB, Uv, Rh7-like 85 aa	n.d.	6	larvae
*Nannochorista philpotti*	Nannochoristidae	3	LwB, Uv, Bl	?	?	larva(e)
*Boreus hyemalis*	Boreidae	4	LwA, LwB, Uv, Bl	n.d.	4	
*Caurinus dectes*	Boreidae	0	—	n.d.	>25	larvae; low number of reads
*Caurinus tlagu*	Boreidae	3	LwB, Uv, Bl	3 males + 2 females	5	initially preserved in EtOH; no ocelli
*Bittacus pilicornis*	Bittacidae	1	LwA 82 aa	n.d.	2	low number of reads
*Harpobittacus australis*	Bittacidae	4	LwA, LwB, Uv, Bl	n.d.	1	
*Chorista australis*	Choristidae	5	LwA, LwB, Uv, Bl, Peropsin	n.d.	1	
*Panorpodes paradoxus*	Panorpodidae	5	LwA, LwB, Uv, Bl	male	4	
*Apteropanorpa evansi*	Apteropanorpidae	2	LwB, Uv 62 aa	n.d.	5	no ocelli
*Apteropanorpa tasmanica*	Apteropanorpidae	3	LwA 69 aa, LwB, Uv	n.d.	1	no ocelli
*Panorpa pryeri*	Panorpidae	5	LwA, LwB, Uv, Bl	male	1	
*Panorpa trizonata*	Panorpidae	10	LwA, LwB, Uv, Bl	male	1	
*Panorpa vulgaris*	Panorpidae	5	LwA, LwB, Uv, Bl	male + female	2	
*Panorpa communis*	Panorpidae	15	LwA, LwB, Uv, Bl, Peropsin	female	1	

The column ‘total opsin hits’ lists the number of opsins retrieved by the semi-automatic search scripts which are included in the Supplementary Material (S2, S3, S4). Since these hits can include highly similar isoforms, the opsin classes listed can be less than the number of hits. For very short fragments the amino-acid (aa) count of the coding region is given. Abbreviations: n.d. = not determined, Bl = blue sensitive, LwA = long wavelength sensitive group A, LwB = long wavelength sensitive group B, Rh7 = *Drosophila* rhodopsin 7, Uv = ultraviolet, ? = no information available.

Before assembly the raw read data of *Panorpa communis* and *Xenopsylla cheopis* were quality trimmed using fastq-mcf ^36^. Thereafter *de novo* assembly was performed with IDBA-Tran^37^ using a maximum k-mer length of 115 and otherwise default settings. For *Panorpa pryeri* and *Apteropanorpa tasmanica* the cDNA libraries have been sequenced twice and the contigs from the two respective assemblies have been pooled for further analysis. The assemblies were searched for transcripts encoding opsins using custom Perl scripts for partial automation: first, a hidden Markov model profile was generated from an alignment of visual opsin protein sequences used in a previous study^38^. Transcripts were translated to all six reading frames and candidate sequences identified by HMMER 3.1b2^39^ were subsequently searched using blastp 2.2.31+^40^ against the manually curated UniProtKB/Swiss-Prot database (release 2013–04)^41^. We only included sequences for further analysis if they either had a HMMER search E-value equal or better than 1E-100 or if the keyword ‘opsin’ was present in their blast description. Candidate sequences were aligned using MAFFT^42^ (L-INSI algorithm). Potential opsin sequences with E-values worse than 1E-15 included in these initial alignments were critically inspected: such sequences were only kept for further processing if the best hit of a search against the NCBI nr (non redundant) database was annotated as an opsin and if key structural motifs of opsins were present (for example a conserved lysine needed to form a Schiff base linkage to the chromophore). Sequences with the longest coding regions were manually identified and highly similar sequences were designated as isoforms (maximum of 5 different amino acids within the coding region, often located at the more error prone contig ends). Isoforms were excluded from further analysis (but are included in Supplementary Table S2 and in Supplementary Data S3 and S4 for full transparency). Three sequences included in the further analysis (Table S2) had frameshifts that were corrected using the program HMM-FRAME^43^ with default options and the same HMM profile that was used for the initial HMMER search. Out of these three, only the blue sensitive opsin transcript in *Apteropanorpa tasmanica* did not exhibit an ambiguity code at the site of the frameshift, so the underlying cause could either be sequencing error or an actual mutation. All identified opsin sequences are supplied as Supplementary material (S2–5).

Salmon 0.7.2^44^ was used to quantify relative transcript abundance (quasi-mapping mode, k-mer length 31). A subset of eight mecopteran species was selected for this analysis based on the presence of all four visual opsins.

A number of opsin sequences from selected additional taxa (Table S1), belonging to different known opsin classes^8,12,38^, was added to the final set of opsin amino acid sequences. After alignment with MAFFT (L-INSI) the 5′ and 3′ untranslated regions were trimmed. To infer phylogenetic trees three different programs were used: IQ-TREE 1.6^45^, RAxML 8.2.0^46^ and ExaBayes 1.4.1^47^. To test for model fit IQ-TREE was run with the -mfp (Model Finder Plus^48^) option, explicitly including a general time reversible (GTR) substitution model using the option -mset GTR20. An LG matrix with empirical base frequencies and 6 free rate categories (LG + F + R6) fits the data best according to the Bayesian information criterion. Under the Akaike information criterion a GTR model with empirical base frequencies and six rate categories is preferred (GTR20 + F + R6). The LG model was used for the subsequent IQ-TREE maximum likelihood (ML) analyses together with options to perform a standard bootstrap analysis (500 replicates) and an ultrafast bootstrap analysis (UFBoot^49^, 2000 replicates). Since trials indicated that setting the -numstop option to 500 and the -pers option to 0.3 yields better trees than with the defaults, we used these settings for the final analysis.

RAxML was utilised to reconstruct a ML gene tree of the dataset using the rapid hill climbing algorithm under the GAMMA model with the GTR substitution matrix. Using this model the program found trees with better ML than IQ-TREE with the GTR20 model. Bootstrap support was inferred using the implemented rapid bootstrap algorithm^50^. The number of bootstrapping replicates (549) was automatically determined by RAxML, using the “bootstop” criterion that checks for convergence of bootstrap replicates on the fly^51^, using the extended majority rule consensus convergence criterion (autoMRE) and the default value of −B 0.03.

For Bayesian tree inference with ExaBayes we ran 1030000 generations in four runs with two Metropolis-coupled chains each. The GTR model and random starting trees were used. An average deviation of split frequencies of 5% was set as a criterion for topological convergence and the final average potential scale reduction factor (PSRF) was 1.02 ± 0.03 and the average effective sampling size (ESS) 180 ± 98. An extended majority rule consensus tree was built discarding the first 25% of samples.

Together with the added previously characterized opsins (e.g. from *Drosophila*), the opsin gene trees were used to assign each siphonapteran and mecopteran opsin to a peak absorbance class (also referred to as predicted spectral sensitivity). The distinction between visual and non-visual opsins in Mecoptera and Siphonaptera as well is based solely on sequence similarity with known physiologically characterized opsins.

## Results

**Figure 1:**
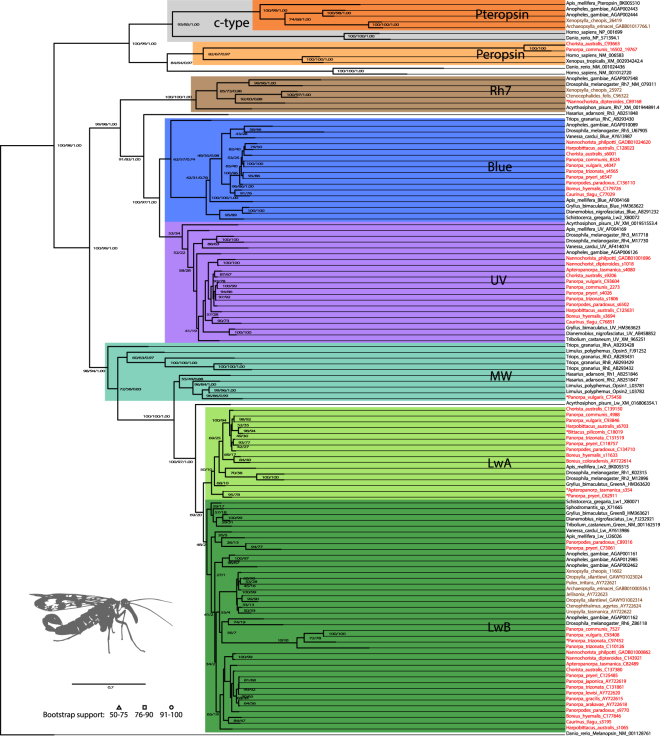
Opsin occurrence in Mecoptera. Best maximum likelihood tree annotated with ultrafast bootstrap support (first number; 2000 replicates) and non-parametric slow bootstrap support (second number; 500 replicates) inferred by IQ-TREE 1.6. A third support value when inferred with the Bayesian approach (ExaBayes, see Supplementary Fig. S6) represents posterior probabilities. The tree was rooted with a *Danio rerio* melanopsin sequence. Visual opsins are highlighted in colours indicating their predicted peak absorbance. Sequences shorter than 100 amino acids included to calculate this tree, are marked by asterisks. Mecopteran and siphonapteran sequence names are highlighted in red and brown, respectively. Appended codes represent NCBI accession numbers (also available in Table S1) or contig and scaffold numbers of the assemblies (Table S2). Abbreviations: Blue = blue sensitive, c = ciliary, LwA = long wavelength sensitive group A, LwB = long wavelength sensitive group B, MW = arthropod medium wavelength sensitive, Rh7 = rhodopsin 7-like opsin. Full sequences and the alignment can be found in the Supplementary Material (S3, S4, S5).

Results from screening the transcriptomes of 15 Mecoptera and six Siphonaptera species are shown in Table 1. In the majority of investigated mecopteran transcriptomes four visual opsins are present (Table 1, Fig. 1, Supplementary Material S2, S3, S4, S5 and Supplementary Fig. S6): LwA = long wavelength sensitive A, LwB = long wavelength sensitive B, Blue = blue sensitive and UV = ultraviolet sensitive. The ocelli-less species *Caurinus tlagu* potentially lacks LwA while in the likewise ocelli-less *Apteropanorpa* species no Blue and only a very short potential LwA candidate were recovered. LwA could not be found in the sequenced *Nannochorista* larvae. Neither blue or UV sensitive opsins were detected in all examined Siphonaptera. Apart from the mentioned visual opsins, further opsin classes, with less well understood function, occur: peropsins in *Chorista australis* and *Panorpa communis*, Rh7-like opsins in *Nannochorista dipteroides*. In the studied Siphonaptera we found a Rh7-like opsin in *Ctenocephalides felis* and *Xenopsylla cheopis*. Pteropsin transcripts (also known as encephalopsin or OPN3-like opsins) are present in the *Xenopsylla cheopis* and *Archaeopsylla erinacei* data.

Apart from terminal regions with low signal-to-noise ratio, the Bayesian tree (ExaBayes, Supplementary Fig. S6) was topologically consistent in deep major nodes with the topology inferred by the ML approach (RAxML, Supplementary Fig. S6; IQ-TREE, Fig. 1). A sister group relationship between the LwA and LwB clades is recovered by all three tree reconstruction programs, albeit with weak support.

**Figure 2:**
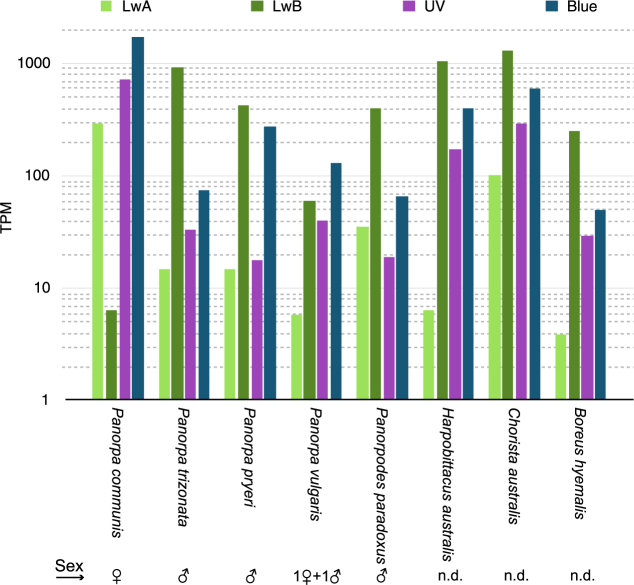
Relative abundance of opsin transcripts in Mecoptera. This plot shows TPM (transcripts per million, logarithmic scale) values calculated by Salmon 0.7.2. In the majority of samples LwB is the most expressed opsin, followed by Blue, UV and LwA. Only in *Panorpa communis* and *Panorpa vulgaris*, the most abundant transcript is Blue. In the only female sequenced, *P. communis*, LwA expression is much stronger than LwB expression. The sex of the investigated specimens is indicated by the pictograms below the figure. Abbreviations: Blue = blue sensitive, LwA = long wavelength sensitive group A, LwB = long wavelength sensitive group B, MW = arthropod medium wavelength sensitive, n.d. = not determined, UV = ultraviolet.

A comparison of the visual opsin transcript abundance (Fig. 2) shows that LwA is expressed less (up to 100-fold) than LwB – latter generally shows the highest relative expression. Only in the female *Panorpa communis* specimen, Blue shows the highest expression and LwA expression is approximately 30 times higher than LwB expression. In the *Panorpa vulgaris* data, which is a 1:1 mixture of male and female tissue^29^, Blue expression is elevated as well. Due to the influence of diurnal variation of expression, sexual dimorphism and technical biases the present results cannot be considered well supported and an in-depth analysis of opsin expression levels would necessitate a more sophisticated experimental design since sampled data from other sources (NCBI & 1KITE) were originally not designed for such purposes. Nevertheless, these values can give a first impression of the relative opsin expression and may serve as a starting point for further investigations.

## Discussion

With around 500 extant species classified into 9 families^52^, Mecoptera are a species-poor group compared to other mega-diverse holometabolous insect orders. Some Mecoptera, specifically the genus *Panorpa* and Bittacidae (represented in our study by *Harpobittacus*), show complex courtship and mating behaviours:^52,53^ Males secrete pheromones for long-range attraction of females and go on to present nuptial gifts (salivary secretions or dead arthropods) while slowly flapping their wings which exhibit a species specific pattern of black spots and/or stripes^54^. It has been stated^54^ that the vision of *Panorpa* is very coarse based on an assumed interommatidial angle Δ*ϕ* of 1°. However, an interommatidial angle of 6° has been found in the central region of the eye of *Panorpa dubia*^26^. Unless Δ*ϕ* is lower in an acute zone of the eye this means that two individuals of this species can only be 19 mm apart for being able to discern stripes of 2 mm width (under the assumption of equally sized gaps between the stripes). That would limit the use of visual communication in *Panorpa* to short ranges. Whether the predaceous Bittacidae, which can catch flying insects while hanging from vegetation or in mid-air^52^, have eyes with better acuity remains to be investigated.

During courtship many Panorpidae also produce a multimodal vibrational and visual signal by moving their abdomen up and down^54^. The abdominal tip of male *Panorpa* bears the clamp-like notal organ that is reddish in most species and the abdomen itself can be brightly coloured as well. While it seems likely that their visual system enables Mecoptera to detect such signals, colour vision in this group has not been investigated in detail so far. Preliminary electroretinogram recordings^55^ in *Panorpa* showed a peak in the green and a shoulder in the UV region. Apart from that, no electrophysiological data on mecopteran spectral sensitivity or conclusive behavioural studies on colour vision are available.

### Opsins in Mecoptera

The first long wavelength sensitive opsin sequences reported for Mecoptera^28^ represent a mix of LwA (*Boreus coloradensis*) and LwB (*Panorpa* spp.). Recently, the additional presence of blue and UV sensitive opsins has been shown for *Boreus coloradensis*^21^. However, in the same study^21^ only a single long wavelength sensitive opsin is documented in *Boreus coloradensis*, *Panorpa acuminata* and *Panorpa nebulosa*. Our results imply that it is most likely that Manwaring *et al*.^21^ failed to retrieve all opsins due to insufficient sequencing depth or other methodological problems. The latter cannot be ruled out since the data is not publicly available. Furthermore, we can refuse their hypothesis^21^ that only female *Panorpa* express a single green sensitive opsin. In our female *Panorpa communis* we found the same set of visual opsins as in male individuals of *Panorpa trizonata* and *Panorpa pryeri*. The claim that electrophysiological data^55^ would support the presence of a single opsin in panorpids is not valid since the summative nature of electroretinograms is not suitable to detect a minority of blue or UV-sensitive receptor cells against a background of predominantly green sensitive receptor cells. Furthermore, these electroretinograms were restricted to the compound eyes^55^.

Only in mecopteran species lacking ocelli (*Caurinus*^56,57^, Apteropanorpidae^58^, *Nannochorista* larvae^23,59^) LwA and Blue (in *Apteropanorpa*) could not be detected. This could indicate that LwA expression is either very low or confined to the ocelli. In Boreidae, the ocelli are reduced in size^58^, or lack the median ocellus^57^, and a peculiar case of missing ocelli in an individual of a species that normally has ocelli (*Boreus westwoodi*) has been reported^60^. While this could mean that at least some Boreidae are on the verge of losing ocelli, the full set of visual opsins is still expressed in the species examined by us.

Since only one female *Panorpa* was sequenced for this study, we cannot provide a definite conclusion on a possible sexual dimorphism of the relative expression of opsins. However, assuming female *Panorpa vulgaris* show an expression pattern similar to the examined female *Panorpa communis*, the observed pattern in the *Panorpa vulgaris* data (three females and one male pooled, see Fig. 2) could probably be explained by a linear combination of male and female values. Of course, only further experimental work (e.g. qPCR) will be able to definitely resolve this question.

### Evolution of opsins among Holometabola

**Figure 3:**
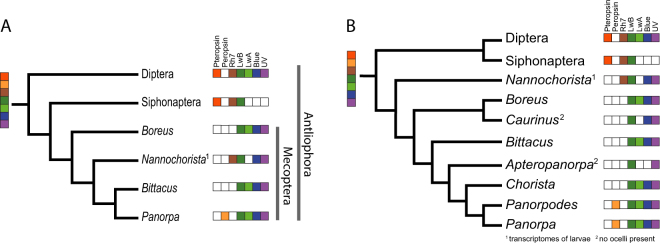
Hypotheses about the phylogenetic relationships within Antliophora and occurrence of opsins within this clade. (**A**) Cladogram based on transcriptomic data^29^. (**B**) Cladogram based on morphological characters^64^ (modified). The pictograms represent the opsins which are reported in this study or are known to be present in Diptera; colours correspond to Fig. 1. The observed distribution suggests that the ancestor of Antliophora possessed at least 7 opsins, as shown by the pictograms at the root of the cladograms.

While the monophyly of the Mecoptera and their internal relationships are not yet unambiguously settled^61^, Diptera and Siphonaptera have been regarded as their closest relatives in almost all traditional and recent analyses^62^. These three orders represent a monophyletic taxon designated Antliophora by Hennig^63^ (Fig. 3). With respect to mecopteran relationships, one particular problem is the phylogenetic position of the peculiarly relictual family Nannochoristidae.

A comprehensive multigene approach^61^ revealed paraphyletic Mecoptera with two major lineages: (Nannochoristidae + (Siphonaptera + Boreidae)) and (Meropidae + ((Choristidae + Apteropanorpidae) + (Panorpidae + (Panorpodidae + Bittacidae)))). The morphological evidence cited supports a sister group relationship between Boreidae and Siphonaptera. More recently, the monophyly of Mecoptera was likewise not supported by a detailed morphological study^64^, and Nannochoristidae was the sister taxon of a clade comprising Diptera and Siphonaptera. Nevertheless, when considering the phylogenetic value of certain morphological characters in combination with molecular evidence, the authors concluded that the paraphyly of Mecoptera is probably artificial. Despite these uncertainties, all the above-mentioned studies recovered Nannochoristidae and Boreidae as more ancestral than Panorpidae. In the only transcriptomic molecular analysis^29^, Mecoptera appeared monophyletic when the amino acid data was analysed, yet support was moderate for the placement of *Nannochorista* within the clade: (*Boreus* + (*Nannochorista* + (*Bittacus* + (*Panorpa*)))).

What can we learn about the evolution of opsins in light of this phylogenetic background? Mapping the results of our study onto a cladogram (Fig. 3) shows that all four visual opsins known for Mecoptera must have been present in the common ancestor of Antliophora. However, this condition is probably much ancestral since it is also found in non-holometabolan insects, such as Orthoptera^38^. Apart from opsins with a known visual function, various other functionally less well-understood opsins (e.g. Rh7-like, pteropsin, arthropsins and peropsins) are present in Pterygota^12,65^. Holometabola initially possessed a pteropsin (reported in Diptera, Lepidoptera, Coleoptera and Hymenoptera^65–67^) which was not found in Mecoptera. So far, the only holometabolans for which peropsins have been reported are Lepidoptera^12^ and Coleoptera (NCBI NW_017259703). Due to the large amount of genomic and transcriptomic data available for Diptera and Hymenoptera, the absence of peropsin in these taxa is fairly well established. Since peropsins are weakly expressed in *Panorpa communis* and *Chorista australis*, it is conceivable that these proteins can be found in other (if not all) Mecoptera, provided the sequencing included both larvae and adults collected at different times of the day. Likewise, other opsins, such as Rh7-like and pteropsins, may be more widespread than the transcriptome data suggest.

To summarize, the common ancestor of Antliophora not only possessed a full set of presumably visual opsins but also pteropsin, peropsin and a Rh7-like opsin. In the course of evolution of this clade, individual opsin genes were either lost in several lineages (Fig. 3) or were not yet detected given the presently available datasets.

## Electronic supplementary material

Supplementary Information

Dataset 1

Dataset 2

Datasets 3 to 5
