# Supplementary material for: Hypothesis on monochromatic vision in scorpionflies questioned by new transcriptomic data
Source: Sci Rep. 2018 Jun 29;8:9872. doi: 10.1038/s41598-018-28098-2 (PMC6026179; doi:10.1038/s41598-018-28098-2)
Supplement: Supplementary file 1 — Supplementary Information [file 41598_2018_28098_MOESM1_ESM.pdf]

Hypothesis on monochromatic vision in scorpionflies questioned by new transcriptomic data

Alexander Böhm[1,*], Karen Meusemann[2,3,4], Bernhard Misof[3], Günther Pass[1]

[1]Department of Integrative Zoology, University of Vienna, Althanstraße 14, 1090 Vienna, Austria
[2]Department of Evolutionary Biology and Ecology, Institute for Biology I, University of Freiburg, Hauptstraße 1, 79104 Freiburg, Germany
[3]Center for Molecular Biodiversity Research, Zoological Research Museum Alexander Koenig, Adenauerallee 160, 53113 Bonn, Germany
[4]Australian National Insect Collection, CSIRO National Research Collections Australia (NRCA), Acton, ACT 2601, Australia
[*]corresponding author; a.boehm@univie.ac.at

Supplementary Information

Supplementary Table S1 (csv format): NCBI Accession numbers and 1KITE library identifiers of all data used

Supplementary Table S2 (csv format): List of all newly identified opsin sequences

FASTA file S3: DNA sequences of all newly identified opsin sequences

FASTA file S4: Protein sequences of all newly identified opsin sequences

FASTA file S5: Alignment used for tree reconstruction

Supplementary Figure S6: Comparison of Bayesian and maximum likelihood opsin gene trees.

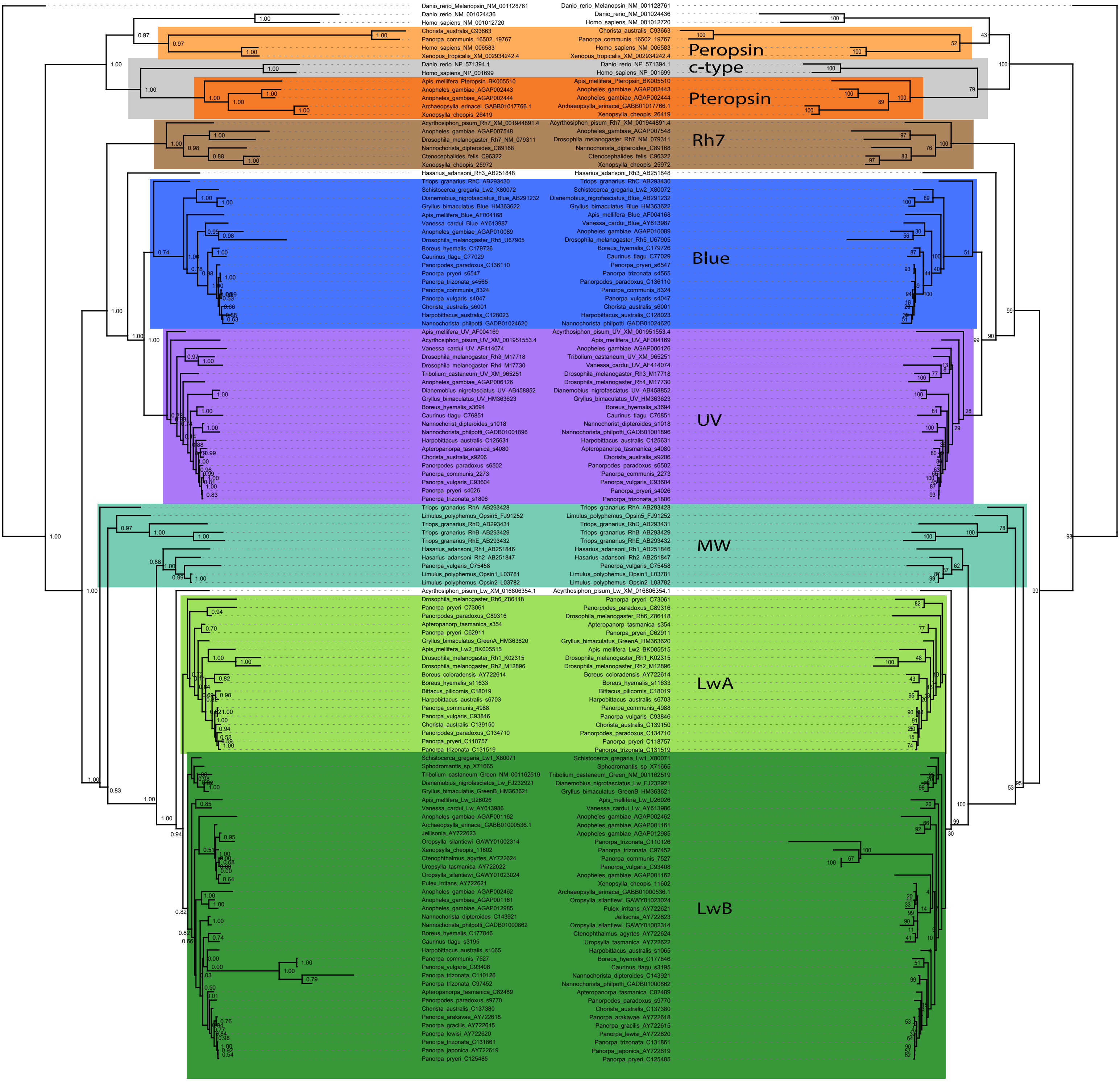

Supplementary Figure S6: **Comparison between Bayesian and maximum likelihood opsin gene trees.** The left tree has been computed by ExaBayes, the right one by RAxML, both using the GTR amino acid substitution model. The shown values represent posterior probabilities and rapid bootstrap support, respectively. Differences only occur within the predicted spectral clades indicated by the coloured overlays. Abbreviations: Blue = blue sensitive, c = ciliary, LwA = long wavelength sensitive group A, LwB = long wavelength sensitive group B, MW = arthropod medium wavelength sensitive, Rh7 = rhodopsin 7-like opsin.
